# Growth hormone-secreting adenoma with lack of retinoblastoma protein expression

**DOI:** 10.1210/jcemcr/luag146

**Published:** 2026-06-03

**Authors:** Roberto Salvatori, Gary L Gallia, Calixto-Hope G Lucas

**Affiliations:** Division of Endocrinology Metabolism and Diabetes, Department of Medicine, Johns Hopkins University School of Medicine, Baltimore, MD 21287, USA; Pituitary Center, Johns Hopkins University School of Medicine, Baltimore, MD 21287, USA; Pituitary Center, Johns Hopkins University School of Medicine, Baltimore, MD 21287, USA; Department of Neurosurgery, Johns Hopkins University School of Medicine, Baltimore, MD 21287, USA; Pituitary Center, Johns Hopkins University School of Medicine, Baltimore, MD 21287, USA; Department of Pathology, Johns Hopkins University School of Medicine, Baltimore, MD 21287, USA

**Keywords:** retinoblastoma, somatotropinoma, immunohistochemistry, tumor suppressor

## Abstract

Retinoblastoma protein (Rb) is a tumor suppressor whose inactivation causes retinoblastomas, pineoblastomas, and possibly other central nervous system tumors. Although mice with *Rb1* gene inactivation invariably develop pituitary adenomas, and some evidence suggests that a subset of pituitary adenomas have lack or reduced expression of Rb protein, to date no evidence has been reported that patients with germline mutations in the *RB1* gene (that encodes for Rb protein) are at risk of developing pituitary adenomas. Here, we report the case of a patient with childhood (age 1 year) onset retinoblastoma because of a germline pathogenic variant of the *RB1* gene who presented with a somatotropinoma diagnosed at a young age (20) and whose pituitary adenoma cells showed loss of expression of Rb protein by immunohistochemistry, suggesting a role of Rb in preventing the development of pituitary adenomas.

## Introduction

The retinoblastoma protein (Rb), encoded by the *RB1* gene (OMIM #180200) is an important regulator of cell duplication, preventing cell proliferation by inhibiting cell-cycle progression from G1 to S phase [1]. *RB1* was the first identified tumor suppressor gene, named for its crucial role in retinoblastoma oncogenesis [2]. Some central nervous system neoplasms harbor inactivating *RB1* variants as oncogenic drivers, including pineoblastoma [3] and glioblastoma [4]. Although mice heterozygous for germline mutation of the *Rb1* gene invariably develop pituitary adenomas arising from the gland's pars intermedia [5], loss of heterozygosity of *RB1* was not observed in 34 human pituitary adenomas [6]. However, a role for loss of Rb function in the development of pituitary adenoma has been suggested by the finding that 8 of 30 (26%) presented methylation of the promoter associated with loss of Rb expression, not seen in normal pituitary glands [7]. Of these 8, 3 were somatotropinomas. Two additional adenomas (both somatotropinomas) failed to express any detectable level of Rb protein, showing loss of heterozygosity [7]. In another study using immunohistochemistry, 20% of the cells of somatotropinomas were negative (10%) or had very low labeling (10%) for Rb protein [8]. Despite this, to our knowledge, no report has been published to date of an association between retinoblastoma and pituitary adenoma in the same patient.

## Case presentation

We recently saw a 20-year-old man with history of bilateral retinoblastoma diagnosed at age 1 year, treated with enucleation of 1 eye and systemic chemotherapy, cryopexy, and thermotherapy of the contralateral eye, who was diagnosed with a 9-mm pituitary adenoma at age 20 years by magnetic resonance imaging obtained during workup of headaches.

## Diagnostic assessment

His examination showed a stature of 195 cm (higher than sex-adjusted mid-parental height of 181 cm), but no obvious acromegaly facial features. He reported having 2 male paternal cousins with similar stature. His serum insulin-like growth factor I was elevated at 738 ng/mL (96.4 nmol/L) (age-adjusted reference range 83-456 ng/mL, 10.9-59.6 nmol/L). Serum prolactin was borderline elevated at 20.6 ng/mL (438 mcIU/mL) (reference values 3.0-14.7 ng/mL, 63.8-312.8 mcIU/mL). Serum GH nadir after a 75-g oral glucose challenge was 2.1 ng/mL (6.3 mIU/mL), confirming the diagnosis of GH-secreting adenoma. Genetic testing from leukocytes showed a heterozygous disease-causing *RB1* variant (c.1981C>T; p.Arg661Trp; NM_000321) in exon 20, consistent with a diagnosis of hereditary retinoblastoma. This pathogenic variant has been reported as having incomplete penetrance for the development of retinoblastomas [9].

## Treatment

The patient underwent successful adenomectomy via a transsphenoidal route, and pathology confirmed a pituitary adenoma with immunostains positive for pituitary-specific positive transcription factor 1 (PIT1), GH, and prolactin, but negative for ACTH and steroidogenic factor-1.

**Figure 1:**
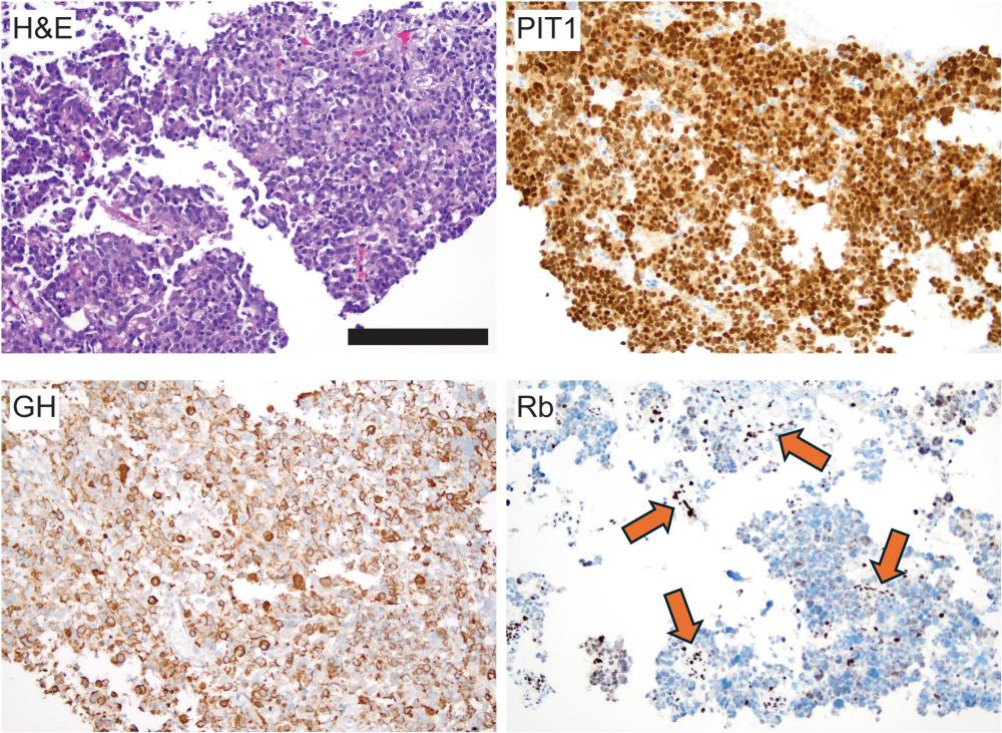
Representative histologic findings from surgical resection specimen of a somatotroph adenoma arising in the setting of “familial retinoblastoma syndrome” (all images ×200 magnification, scale bar 200 µm). Histopathologic examination revealed a tumor composed of a monotonous population of irregular cells with round nuclei, fine chromatin, and prominent nucleoli arranged in sheets, consistent with a pituitary adenoma (pituitary neuroendocrine tumor; PitNET). The tumor cells were diffusely immunoreactive for pituitary-specific positive transcription factor 1 (PIT) with extensive positivity for GH, supporting the somatotroph adenoma diagnosis. Immunohistochemical expression of Rb was significantly reduced in tumor cells while retained in endothelial cells (arrows).

Based on the previously mentioned mouse and human data, we hypothesized that Rb protein expression may have been lost in the adenoma of this patient. To this end, Rb immunostaining (Cell Signaling, cat#9309T, 1:5000) was performed on the pathology sample, and absent immunohistochemical expression of Rb was observed in adenoma cells, whereas it was retained in endothelial cells (Fig. 1). The Rb antibody recognizes an epitope corresponding to a region surrounding p.His890. The reported pathogenic germline variant is a missense mutation involving codon 661 that replaces arginine, which is basic and polar, with tryptophan, which is neutral and slightly polar. Although this results in a conformational change that reduces the functional capacity of the Rb protein, we hypothesize that this mutation also results in a structural change that reduces immunoreactivity for the Rb antibody.

## Outcome and follow up

Postoperatively, his headaches resolved. Serum GH became unmeasurable, and 3.5 months after surgery, his serum insulin-like growth factor I was normal at 236 ng/mL (30.9 nmol/L).

## Discussion

Germline mutations in several genes have been associated with the development of GH-secreting pituitary adenomas: aryl hydrocarbon receptor-associated protein (*AIP*), protein kinase cAMP-dependent type I regulatory subunit alpha (*PRKAR1A*), G-protein coupled receptor 101 (*GPR101*), guanine nucleotide-binding protein alpha stimulating activity polypeptide (*GNAS*), menin (*MEN1*), cyclin-dependent kinase inhibitor 1B (*CDKN1B*), succinate dehydrogenase complex genes (*SDHx*), and MYC-associated transcriptional regulator X (*MAX*) [10]. Although children with retinoblastoma have a high risk of developing second cancers such as soft-tissue sarcoma, osteosarcomas, melanoma, Hodgkin disease, leiomyosarcoma and prostate, breast, brain, lung, and buccal cavity (salivary gland and tongue) cancers [11], to date *RB1* has not been included in the list of genes predisposing to the development of pituitary adenomas. However, evidence of lack of Rb expression in pituitary adenomas has been reported in a subset of these tumors [7, 8]. Our current observation suggests that this patient's *RB1* gene may have undergone loss of heterozygosity or promoter methylation and contributed to the development of the adenoma. Interestingly, in mice with defective *Rb1,* these adenomas develop from the pars intermedia, a distinct lobe situated between the anterior and posterior pituitary, composed of 10 to 15 layers of densely arranged, pigment-stimulating melanotrophic cells that produce pro-opiomelanocortin-derived peptides. The rarity of reported pituitary adenomas with Rb inactivation in humans may be due to the fact that the pars intermedia is rudimentary and represents the vestigial posterior limb of Rathke's pouch [12]. However, while this zone may also contain secretory cells able to secrete alpha- or beta-endorphin, ACTH and alpha-melanocyte stimulating hormone, no evidence of GH secretory cells in this area has been reported [12].

In summary, we report what we believe is the first case of GH-secreting adenoma in a patient with a germline pathogenic *RB1* variant, and we show immunohistochemical evidence of Rb protein loss of expression in adenoma cells, suggesting a pathogenic role in adenoma development.

## Learning points

- Somatotropinomas can occur in several inherited genetic syndromes.
- An association between somatotropinomas and retinoblastoma has not been previously reported.
- *RB1* pathogenic variants may predispose to the development of somatotropinomas.

## Contributors

All authors made individual contributions to authorship. R.S. and G.L.G. were involved in the diagnosis and management of this patient. C.H.G.L.J. was responsible for histopathology sections and preparation of histology images. G.L.G. was responsible for the patient's surgery. All authors reviewed and approved the final draft.

## Data Availability

Data sharing is not applicable to this article as no datasets were generated or analyzed during the current study.
